# Nephroprotective effects of GLP-1 receptor agonists: where do we stand?

**DOI:** 10.1007/s40620-020-00738-9

**Published:** 2020-04-30

**Authors:** Charlotte M. Mosterd, Petter Bjornstad, Daniël H. van Raalte

**Affiliations:** 1 Diabetes Center, Department of Internal Medicine, Amsterdam University Medical Centers, Location VUMC, De Boelelaan 1117, 1081 HV Amsterdam, The Netherlands; 2 Section of Endocrinology, Department of Pediatrics and Division of Nephrology, Department of Medicine, University of Colorado School of Medicine, Aurora, CO USA

**Keywords:** GLP-1 receptor agonists, Diabetic kidney disease, Albuminuria, Blood pressure, Incretin-based therapies

## Abstract

Glucagon-like peptide (GLP)-1 receptor agonists are the cornerstone in the treatment of hyperglycemia in many people suffering from type 2 diabetes (T2D). These drugs have potent glucose-lowering actions and, additionally, lower body weight through satiety induction while reducing blood pressure and dyslipidemia. Partly through these actions, GLP-1 receptor agonism was shown to reduce cardiovascular disease (CVD) in people with T2D with previous CVD or at high-risk thereof. In these cardiovascular safety trials, in secondary or exploratory analyses, GLP-1 receptor agonists were also shown to reduce macro-albuminuria, an accepted surrogate marker for diabetic kidney disease (DKD), a condition that still represents a major unmet medical need. In this review we will discuss the evidence which suggests renoprotection induced by GLP-1 receptor agonists and the potential mechanisms that may be involved. These include mitigation of hyperglycemia, overweight and insulin resistance, systemic and glomerular hypertension, dyslipidemia, sodium retention, inflammation and renal hypoxia. The recently initiated large-sized FLOW trial investigating the effects of semaglutide on hard renal outcomes in patients with DKD will provide clarity whether GLP-1 receptor agonists may reduce the burden of DKD in addition to their other beneficial metabolic and cardiovascular effects.

## Introduction

The prevalence of people living with type 2 diabetes (T2D) has increased to over 450 million worldwide and is expected to increase further in the next decades [1]. The life expectancy of people with T2D is reduced due to increased mortality from cardiovascular disease (CVD). The strongest risk factor for CVD and mortality in T2D is diabetic kidney disease (DKD) and DKD has been proposed to account for the majority of CVD risk observed in people with diabetes. In fact, although diabetes is the main cause for end-stage kidney disease (ESKD) worldwide, many people with T2D die from CVD before reaching ESKD [2]. DKD afflicts 20–40% of people with T2D, and manifest as increased urinary albumin excretion, impaired GFR or a combination of both [3]. It is therefore clear that DKD represent a major unmet medical need. Prevention and slowing down of DKD currently focuses on risk factor mitigation, including dietary modification (e.g. low protein, salt intake and caloric targets), encouraging a healthy lifestyle with sufficient exercise and smoking cessation. Pharmacologically, lowering hyperglycemia and hyperlipidemia as well as achieving tight blood pressure control is pursued. For the latter, the renin–angiotensin–aldosterone system inhibitors (RAS) blockers are used, given their renoprotective efficacy beyond blood pressure lowering per se. Nevertheless, as demonstrated in the STENO-2 trial, residual renal and risk and overall mortality remains high, also due to the fact that the different treatment targets are often not reached [4].

Recent years have seen several pharmacological developments that may alter the outlook for people living with DKD. First, the class of sodium glucose cotransporter-2 (SGLT2) inhibitors was recently introduced. These agents lower blood glucose levels by blocking glucose reabsorption in the proximal tubule. Due to (temporarily) reduced sodium reuptake while continuously promoting glucosuria, these drugs induce a reduction in GFR likely via activation of tubuloglomerular feedback (TGF) that may represent reduced intraglomerular pressure [5]. In cardiovascular outcome trials (CVOTs) such as Empagliflozin Cardiovascular Outcome Event Trial in Type 2 diabetes Mellitus Patients (EMPA-REG OUTCOME), CANagliflozin cardioVascular Assessment Study (CANVAS) and The Dapagliflozin Effect on Cardiovascular Events–Thrombolysis in Myocardial Infarction 58 (DECLARE-TIMI 58) [6], they reduced the onset and progression of DKD, which was confirmed in the dedicated kidney trial The Canagliflozin and Renal Endpoints in Diabetes with Established Nephropathy Clinical Evaluation (CREDENCE), which showed a canagliflozin-induced risk reduction for ESKD in people with DKD by 32% [7]. Of note, SGLT2 inhibitors also reduce cardiovascular mortality or hospitalization for heart failure, thus showing benefits across the cardiorenal axis. Second, over a decade ago, market access was granted to the first glucagon-like peptide (GLP-1) receptor agonist. This class of drugs are frequently used by diabetologists for their strong glucose-lowering effects, beneficial safety profile as well as for their cardiovascular protection as described below. Emerging data mostly acquired from the CVOT’s conducted for the various GLP-1 receptor agonists now available, also suggest that they may offer renoprotection [8]. In this review, the mechanism of action of GLP-1 receptor agonists on glucose metabolism and on the kidney are discussed as well as the (pre)clinical data that currently are suggestive of renoprotection, and potentially involved pathways.

## Diabetic kidney disease—a heterogenous disease with shared risk factors

DKD, especially in people with T2D, is heterogenous in nature and driven by several risk factors. These include but are likely not limited to chronic hyperglycemia, hypertension, dyslipidemia, obesity, insulin resistance, sodium retention, neurohormonal activation and low-grade inflammation, all related to the metabolic syndrome [9, 10]. In addition to these above risk factors, another driver of DKD may be glomerular hyperfiltration, i.e. increased GFR that can be manifest at the whole kidney level (GFR > 135 mL/min/1.73 m^2^) in adolescents and young adults with T2D [11] or as increased single-nephron GFR in adults or older people with T2D that have reduced nephron mass; both conditions are thought to represent a state of glomerular hypertension that damages the glomerular capsule [12]. Finally, the diabetic kidney may be predisposed to chronic tissue hypoxia, in part due to impaired renal perfusion secondary to hyperglycemia-associated microvascular damage. Additionally, the diabetic kidney has increased oxygen demand due to increased sodium reabsorption and (single-nephron) hyperfiltration via increased Na^+^, K^+^ ATPase activity at the basolateral surface of the tubules [13]. The hyperglycemia-related increased proximal tubular sodium reabsorption results in lower sodium levels at the level of the macula densa increasing GFR via inhibiting TGF, further augmenting the amount of filtered and reabsorbed sodium with increased ATP demands. ATP generation on the other hand may be inadequate due to impaired substrate utilization due to insulin resistance and mitochondrial dysfunction [14]. The net effect of the mismatch between increased ATP demand and decreased ATP generation is increased renal oxygen consumption, ultimately rendering the kidney susceptible to renal hypoxia [15]. Hypoxia predisposes to structural defects including fibrosis and nephron loss. Analyses of renal biopsies obtained from T2D patients support a multifactorial pathophysiology as the structural damage observed varies. As such, glomerular lesions occur which include glomerular basement membrane thickening, podocyte effacement and loss, mesangial expansion, epithelial–mesenchymal transition EMT and fibrosis, collagen deposition, damage to the capillary architecture and an influx of inflammatory cells including macrophages. Beyond glomerular damage, tubular atrophy and tubulointerstitial inflammation have also been demonstrated, while vascular lesions include arterial hyalinosis [16].

As a drug class, the GLP-1 receptor agonists possess properties that could translate to improved renal energetics including its effects on substrate metabolism. It remains unclear, however, whether such effects mitigate hypoxia risk and ultimately fibrosis and nephron loss as described below.

## Glucagon-like peptide (GLP)-1: the incretin effect and gut-renal axis

GLP-1 was discovered to be one of the key hormones to underlie the so-called incretin effect, i.e. an augmented insulin response to an oral glucose load compared with intravenous (IV) glucose administration. GLP-1 is secreted from L cells in the small intestine upon food stimuli including carbohydrates, lipids, proteins, amino acids, bile acids and short-chain fatty acids. GLP-1 has a short half-life of ~ 2–3 min due to rapid inactivation by the enzyme dipeptidyl peptidase (DPP)-4. Through neural pathways as well as via receptor-mediated effects, GLP-1 reduces postprandial hyperglycemia by stimulation of insulin secretion, suppression of glucagon release, inhibition of gastric emptying and small intestinal peristalsis, and induction of satiety which limits meal size [17]. The glucometabolic effects of GLP-1 have been reviewed extensively elsewhere [18] and are not the focus of the current review.

In addition to its glucoregulatory effects, GLP-1 was shown to interact with other organs as well, which is not surprising given the widespread expression of GLP-1 receptors in the human body. As such, GLP-1 has been linked to the kidney as a feed-forward sensor that is part of a so-called gut-renal axis [19]. In non-diabetic mice, GLP-1 infusion increased GFR by reducing preglomerular resistance [20], an observation that could potentially explain the physiological increase in GFR observed in the postprandial compared to the fasting state, although human studies infusing GLP-1 peptide could not replicate this observation [21]. Meal-induced increments in GFR could serve to enhance the elimination of nitrogen waste products as well as electrolytes such sodium and potassium. Indeed, both in rodents and humans, GLP-1 infusion increased fractional sodium excretion [20], which has been linked to the finding that sodium is more rapidly excreted following an oral versus equivalent IV sodium load in a number of experimental studies in healthy individuals, independent of factors such as atrial natriuretic peptide (ANP) and aldosterone [22]. GLP-1 may induce sodium excretion by blocking sodium reabsorption in the proximal tubule through inhibition of sodium-hydrogen exchanger isoform 3 (NHE3) activity [23], as no GLP-1 receptors appear to be present in human kidneys outside of the renal vasculature [19].

In people with T2D, the incretin effect is severely attenuated, despite similar levels of circulating GLP-1. However, a dose-dependent insulinotropic and glucagonostatic response is preserved in response to exogenous GLP-1 treatment and 6-week subcutaneous GLP-1 infusion was shown to improve HbA1c levels by 1.3% [18]. However, due to its indicated short half-live, GLP-1 peptide is an unattractive therapeutic agent on itself, which stimulated the quest for GLP-1 based compounds with better bioavailability.

## Development of GLP-1 receptor agonists

The most important strategy to develop compounds with extended in vivo half-life of GLP-1 was by making DPP-4 resistant GLP-1 receptor agonists. Over the last decade and a half, several (injectable and most recent an oral) GLP-1 receptor agonist formulations have been introduced for the management of hyperglycemia in people with T2D [24].

The first molecules that were developed were based on an exendin-4 backbone which was isolated from the saliva of the Gila monster (*Heloderma suspectum*) which shares 53% homology with GLP-1. Exenatide (twice-daily) and lixisenatide are GLP-1 receptor agonists based on exendin-4 that overcome cleavage by DPP-4, however, their half remains short with ~ 2–4 h due to renal elimination. They are classified as short-acting or postprandial GLP-1 receptor agonists due to their glucose-lowering effects in the postprandial state [24]. Second generation GLP-1 receptor agonists that were developed have more similarity with human GLP-1 and are linked to larger carrier molecules to limit renal elimination. These include the once-daily GLP-1 receptor agonist liraglutide and the once-weekly compounds albiglutide, dulaglutide, semaglutide and a long-acting release formulation of exenatide (formulated within biodegradable polymeric microspheres). The long-acting GLP-1 receptor agonists have strong effects on insulin secretion and fasting glucose levels, but lose their postprandial glucose-lowering properties due to receptor tachyphylaxis. GLP-1 receptor agonists lower HbA1c by 1–1.5% but this may be dependent of the type of GLP-1 receptor agonist prescribed, the dosage, baseline HbA1c levels and glucose-lowering co-medication [24]. Due to their glucose-lowering efficacy as well as other beneficial effects on blood pressure, body weight and lipid metabolism, they have taken a central place in the management of hyperglycemia in people with T2D. Based on studies in people with chronic kidney disease (CKD), GLP-1 receptor agonists were shown to be safe (no increased risk for acute kidney injury (AKI) was observed) and efficacious also at lower eGFR ranges [25]. Liraglutide is currently approved for people with T2D and an eGFR > 15 mL/min/1.73 m^2^, and besides metformin and insulin is the only diabetes medication approved by FDA for adolescents with youth-onset T2D [26].

## GLP-1 receptor agonists and renal outcomes

Based on the US FDA industry guidance issued in 2008, all new glucose-lowering agents were required to perform a large-sized placebo-controlled cardiovascular outcome/safety trial, usually conducted in people with T2D with pre-existent or at high-risk of developing CVD. At present, seven trials have currently been reported for GLP-1 receptor agonists which include ELIXA (Evaluation of LIXisenatide in Acute coronary syndrome; lixisenatide), LEADER (Liraglutide Effects and Action in Diabetes: Evaluation of cardiovascular outcome Results; liraglutide), SUSTAIN-6 (Trial to Evaluate Cardiovascular and Other Long-term Outcomes With Semaglutide in Subjects With Type 2 Diabetes; subcutaneous semaglutide), EXSCEL (EXenatide Study of Cardiovascular Event Lowering; exenatide once weekly), REWIND (Researching Cardiovascular Events With a Weekly Incretin in Diabetes; dulaglutide), HARMONY (Albiglutide and cardiovascular outcomes in patients with T2D and cardiovascular disease; albiglutide) and PIONEER-6 (oral semaglutide) [8]. The primary outcomes of these trials were 3-point major adverse cardiovascular event (3-MACE) and the studies have together provided a wealth of data that, amongst others, have changed clinical guidelines. We refer to a recent systematic review and meta-analysis for details regarding the effects of GLP-1 receptor agonists on cardiovascular outcomes [8]. In short, despite some heterogeneity across the trials, GLP-1 receptor agonists were shown to reduce the hazard ratio’s [95%CI] for 3-MACE by 12 [6–18]%, cardiovascular death by 12 [4–19]%, myocardial infarction by 9 [0–16]% and stroke by 16 {7–24]%. In these trials, data on renal outcomes were also collected (Table 1), although not all trials had this as a prespecified outcome, and therefore the data should be interpreted with caution.

**Table 1:**
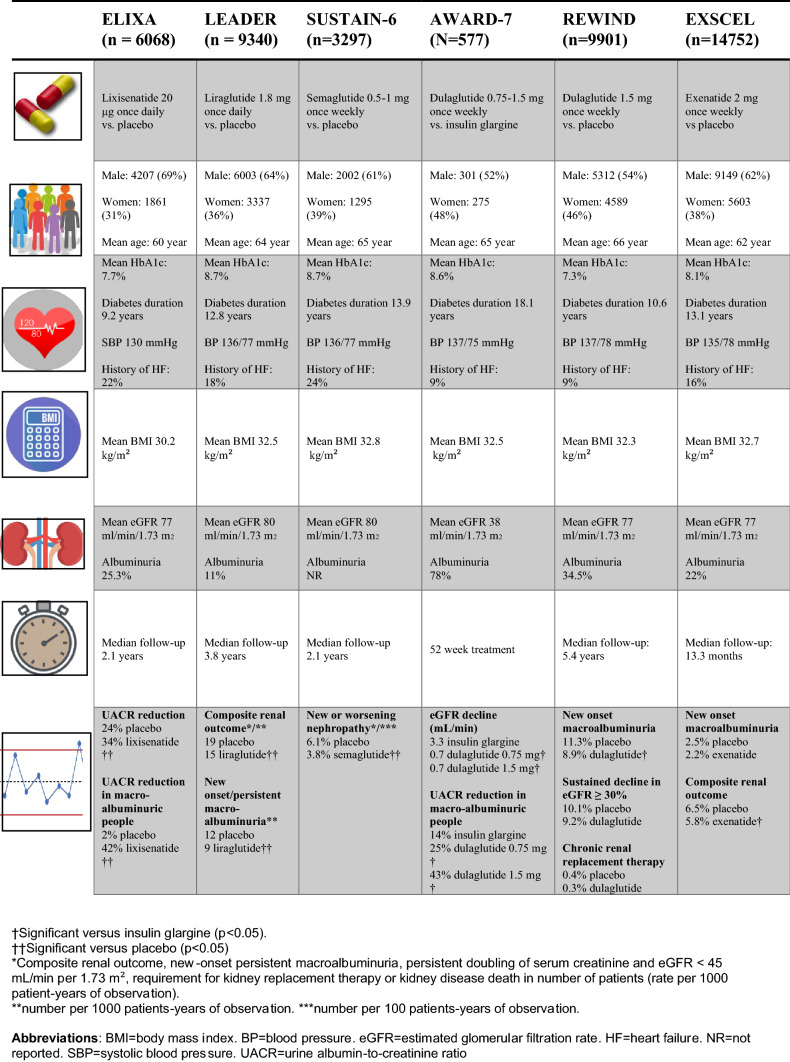
Renal outcomes in six major cardiovascular safety trials assessing glucagon-like peptide-1 receptor agonists in people with type 2 diabetes

In ELIXA, the effects of lixisenatide versus placebo in 6,026 people with T2D and a recent acute coronary event were investigated. Lixisenatide reduced urinary albumin-to-creatinine ratio (UACR) versus placebo at 108 weeks of follow-up by 10% (p = 0.004). Correction for small differences in glycemic control during the trial diminished significance indicating some glucose-dependency [27]. In a separate analysis, lixisenatide reduced incident macroalbuminuria, while adjustments for baseline characteristics such as age, blood pressure and eGFR, as well as on trial HbA1c, blood pressure and weight, did not significantly attenuate the association [28]. In a sub-analysis in people with albuminuria at baseline, lixisenatide reduced UACR by 21% (in people with microalbuminuria) 39% (in people with macroalbuminuria). Lixisenatide did not change eGFR decline, and incidences of hard renal endpoints were too low to be meaningfully analyzed [28].

Subsequently, LEADER and SUSTAIN-6 were reported, which investigated respectively liraglutide and semaglutide vs. placebo in 9340 and 3297 people with T2D and high cardiovascular risk. LEADER [29] and SUSTAIN-6 [30] reported a prespecified renal composite of new onset or persistent macroalbuminuria, persistent doubling of serum creatinine and eGFR ≤ 45 mL/min/1.73 m^2^, need for renal replacement therapy (RRT), and renal death. Liraglutide reduced this renal outcome by 22% after 3.8 years follow up, whereas semaglutide showed a reduction of 36% after 2.1 years. Importantly, these reductions were solely driven by reductions in macroalbuminuria without effects on hard renal endpoints. As liraglutide and semaglutide induced stronger HbA1c reductions versus placebo then lixisenatide, it was important that the authors showed in reply to a letter to the editor that the anti-albuminuric effects were independent of glucose-lowering in post-hoc analyses. Regarding eGFR trajectories, both drugs induced a slightly slower decline in eGFR compared to the placebo groups, which was more pronounced in people with moderate (eGFR 30–59 mL/min/1.73 m^2^) or severe (eGFR < 30 mL/min/1.73 m^2^) impaired renal function at baseline. In contrast to RAAS blockers and SGLT2 inhibitors however, this higher eGFR at end of treatment was not caused by an initial drop in eGFR with stabilization over time (suggestive of a reduction in glomerular pressure), but rather by an upsurge in eGFR with similar rates of eGFR decline over time versus placebo [29, 30].

In the EXSCEL trial, 14,752 people with T2D and previous CVD were exposed to exenatide once weekly or placebo for a median duration of 3.2 years. A prespecified renal endpoint which was driven by incident macroalbuminuria was significantly reduced after adjustments for baseline factors including age, sex, BMI, HbA1c and eGFR. No other endpoints were affected, including eGFR (Table 1). [31].

While the HARMONY outcomes reported no change in eGFR after 16 months of treatment, no other renal outcomes were presented, which also holds true for the recently completed PIONEER-6 trial with oral semaglutide.

Finally, the REWIND trial reported renal outcomes after 5.4 years of dulaglutide treatment versus placebo. In addition to reducing albuminuria, dulaglutide reduced the surrogate renal endpoints of 40% or 50% decline in eGFR (HR 0.70 and 0.56 respectively; p < 0.001 for both). The absolute eGFR differences between the groups by the end of the study were small (0.42 ml/min/1.73 m^2^) and the eGFR slopes did not meaningfully differ after the initial weeks of treatment [32].

## GLP-1 receptor agonist treatment in people with DKD

Limitations of the above-described studies include exploratory renal endpoints with limited adjudication, and small number of participants with baseline DKD. Currently, there are only a few trials that investigated the effects of a GLP-1 receptor agonist in people with DKD, however they focused primarily on glucose-lowering and two were of short duration. In the AWARD-7 trial, the effects of low- and high-dose dulaglutide versus titrated insulin glargine, both added to prandial insulin humulin, was compared in 576 people with T2D and moderate to severe CKD. Compared to insulin glargine, dulaglutide reduced UACR by 39 (10–69) % in patients with macroalbuminuria at baseline [33]. In addition, dulaglutide lead to lower eGFR decline compared with titrated insulin glargine: the cystatin C-based eGFR decline of 52-week dulaglutide high-dose was 0.7 mL/min/1.73 m^2^ and low-dose 0.7 mL/min/1.73 m^2^ vs. 3.3 mL/min/1.73 m^2^ with insulin glargine. Similar to what was observed in the participants with CKD stage 3–4 in LEADER, the differences in eGFR were caused by an initial upsurge in eGFR with similar decline rates over the course of the study. In LiraRenal (liraglutide versus placebo in people with type 2 diabetes and eGFR 30–59 mL/min/1.73 m^2^), liraglutide effective lowered HbA1c and body weight, but did not affect eGFR after 26 weeks of treatment [25]. Similarly, in PIONEER-5 where 324 patients with diabetes and eGFR 30–59 mL/min/1.73 m^2^, oral semaglutide showed safety and efficacy on glycemic endpoints, but did not alter eGFR, although it lowered UACR to a similar extent as the injectable GLP-1 receptor agonists [34].

## Potential mechanisms involved in albuminuria lowering

**Fig. 1:**
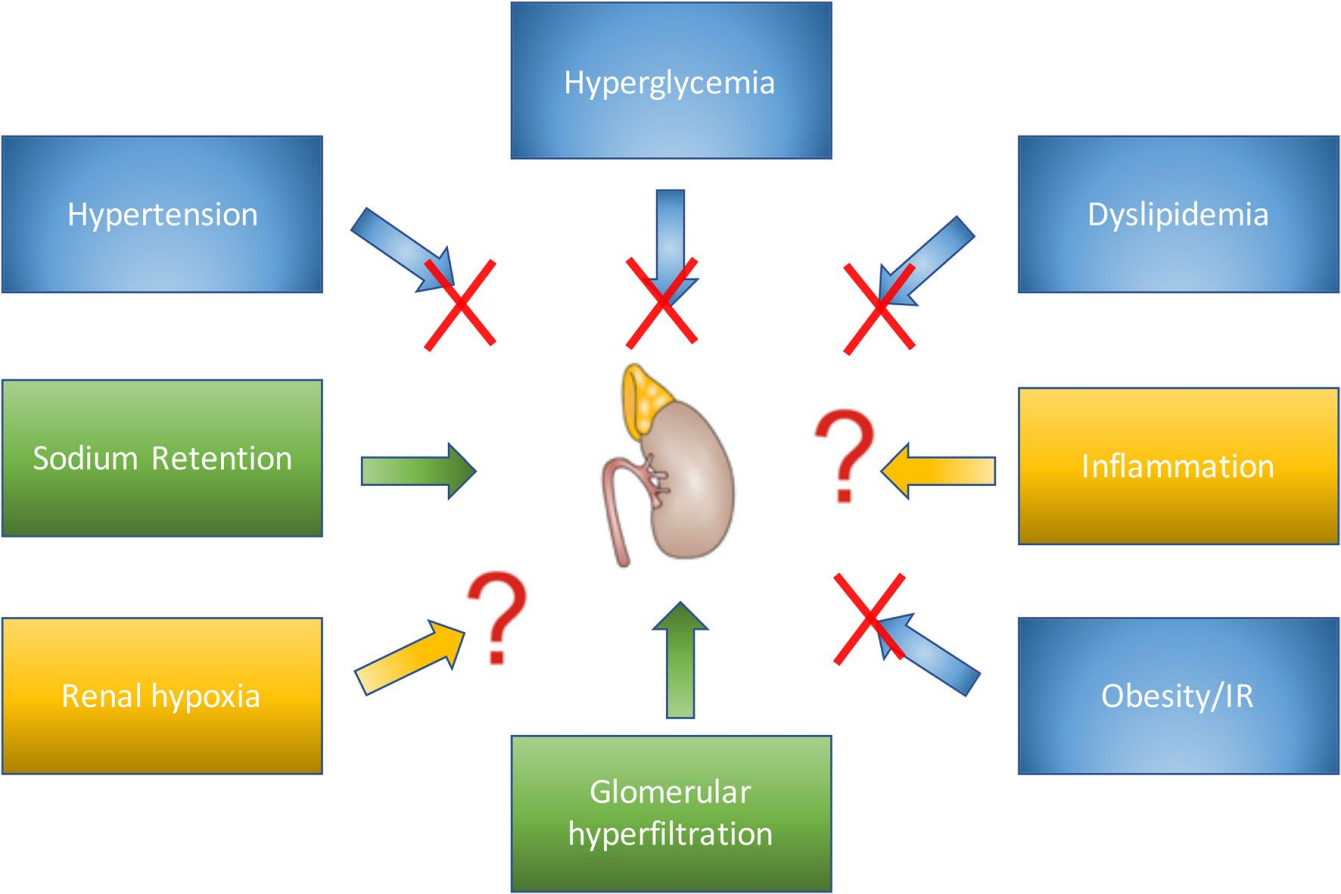
GLP-1 receptor agonists effects on established and presumed drivers of diabetic kidney disease (DKD). In blue boxes, established renal risk factors that are mitigated by GLP-1 receptor agonism (illustrated by a red cross). The risk factors of sodium retention and glomerular hyperfiltration (green boxes) are likely not reduced by GLP-1 receptor agonists. Finally, in yellow boxes, more recently discovered mechanisms of renal damage including inflammation and renal hypoxia are indicated. It is unclear whether GLP-1 receptor agonism modulates these pathways (color figure online)

Several mechanisms have been proposed to explain how GLP-1 receptor agonists may lower albuminuria. These involve classical renal risk factors as well as some novel mediators of DKD. We will address these risk factors/pathways individually below (summarized in Fig. 1). We have started with ‘classic renal risk factors’ and then move on to more novel/experimental pathways.

### Hyperglycemia

Observational studies have clearly showed a causal link between hyperglycemia and DKD incidence, mechanistically caused hyperglycemia-induced glomerular hyperfiltration as well pathways associated with renal glucose toxicity [9]. Long-term intervention trials demonstrated that intensive glucose-lowering versus lenient glucose control reduced DKD incidence. In the United Kingdom Prospective Diabetes study (UKPDS) intensive glucose control reduced the risk of macroalbuminuria by 33%. Similarly, in The Action in Diabetes and Vascular Disease: Preterax and Diamicron MR Controlled Evaluation (ADVANCE) trial, intensive glucose control reduced ESKD by 46 (95% CI 15–66)% [35]. Thus, given the potent glucose-lowering actions of the GLP-1 receptor agonists as described (HbA1c reduction 1–1.5% and even more pronounced for the most recent long-acting GLP-1 receptor agonists), reduction in hyperglycemia may contribute to their strong anti-albuminuric effects. However, post hoc analyses showed only mild changes in the effect size when adjusted for on-trial HbA1c concentrations suggesting additional mechanisms independent of glycemic control.

### Hypertension

Reducing blood pressure has proven to be renoprotective, particularly demonstrated in studies involving RAS blockers [36, 37], although exact blood pressure targets remain debated. Thus, a glucose-lowering agent that also reduces blood pressure is of added benefit. Mechanistically, systemic hypertension may drive DKD by inducing pressure-induced glomerular damage. GLP-1 receptor agonists increase blood pressure acutely, but lower blood pressure after prolonged treatment [19]. The systolic blood pressure reduction is 3–4 mmHg with slight differences observed between the different GLP-1 receptor agonists. The mechanisms by which GLP-1 receptor agonists reduce blood pressure are incompletely understood, but may relate to improvements in insulin sensitivity secondary to weight loss. In addition, inhibition of RAAS activity, improved endothelial function and augmented natriuresis have been proposed. Similar to hyperglycemia, however, controlling for on-trial blood pressure did not significantly alter the reductions in albuminuria induced by GLP-1 receptor agonist treatment in the trials [28].

### Dyslipidemia

Dyslipidemia is a strong risk factor for both CVD and DKD, both with respect to elevated LDL cholesterol, triglycerides and reduced HDL concentrations. Biopsy studies in animals indicated that hyperlipidemia may cause glomerular lipid depositions, an observation that was termed glomerular atherosclerosis [38]. Data from lipid-lowering trials, most notably conduced with statin therapy, were however unable to demonstrate benefits of LDL-lowering on renal outcomes [39, 40]. GLP-1 receptor agonist therapy was shown in a meta-analysis to induce small reductions in the levels of LDL cholesterol, total cholesterol and triglycerides, but not to improve HDL cholesterol levels in comparison to placebo and active comparators [19]. Mechanistically, GLP-1 receptor agonists reduce intestinal chylomicron production and secretion, in addition to potential inhibit on hepatic very-low density lipoprotein (VLDL) production [41]. It is uncertain whether these actions contribute to potential renoprotective effects of GLP-1 receptor agonists.

### Obesity and insulin resistance

Obesity and associated insulin resistance play a crucial role in the development of nearly all metabolic abnormalities associated with T2D. With respect to the kidneys, obesity may predispose to albuminuria and impaired GFR due to the fact that it contributes to many of the established renal risk factors summarized above. Additionally, obesity has been linked to DKD by provoking glomerular hyperfiltration, secondary to changes in sodium retention and increased sympathetic nervous system activity [42]. Intrarenal fat deposition (‘fatty kidney’), in particular fat accumulation in the renal sinus (RSF), has been linked to hypertension [43], renal inflammation and fibrosis, and unfavorable renal hemodynamic function [44]. Intervention trials aimed at weight loss have indeed showed evidence for renoprotection. As such, 4 kg weight loss observed in the landmark life style study LOOK-AHEAD reduced the incidence of very-high-risk chronic kidney disease by 31% at 8 years of follow-up [45], and results from bariatric surgery cohorts have similarly observed renal benefits (reduced hyperfiltration, reduction in UACR) of weight loss [46]. In a recent comparison analysis of the Teen-LABS and TODAY studies, bariatric surgery was shown to attenuate DKD to a substantially greater degree than standard medical therapy in severely obese youth and young adults with T2D [47]. It should be noted, however, that estimated eGFR may be biased in this population where severe weight loss may be accompanied by muscle mass loss, making eGFR that was often reported less reliable. GLP-1 receptor agonist therapy induces weight loss of up to 2 kg compared with placebo and up to 7 kg compared with antihyperglycemic drug classes that are associated with weight gain, most notably insulin therapy [19]. It should be noted that there is much variation with respect to individual responses and the different GLP-1 receptor agonists, with the long-acting compounds inducing stronger body weight reductions. Nevertheless, correction for weight loss in the CVOT’s and AWARD-7 study only partly explained the observed albuminuria reduction. This BMI-correction does not rule out a role for reducing local fat deposition, either in close proximity to kidney tissue or located in the perivascular space. In preclinical studies, GLP-1 receptor treatment was able to reduce renal fat content and concomitantly reduce inflammation and formation of reactive oxygen species (ROS) [48]. However, such mechanisms still need to be interrogated further in clinical research.

### Sodium retention

Excessive salt intake, characteristic of a Western diet, has been linked to hypertension, CVD, and CKD [49]. This may be particularly true for people with T2D who have greater salt sensitivity, secondary to impaired non-osmotic sodium storage or impaired sodium excretion [50]. The latter is associated with obesity, insulin resistance and exogenous insulin therapy that may drive tubular reabsorption of sodium. Similar to endogenous GLP-1 activity, GLP-1 receptor agonists affect tubular function, as intravenous administration of exenatide induced natriuresis in healthy males and individuals with T2D [51, 52]. The most likely involved tubular transporter mediating the natriuretic effects of GLP-1 is NHE3 which is located at the luminal border of the proximal tubule. Indeed, GLP-1 receptor agonists phosphorylate NHE3 at the protein kinase A consensus sites Ser552 and Ser605, thereby reducing its activity [53]. Acute GLP-1 receptor agonist treatment also enhances renal lithium clearance (a marker of proximal tubular sodium reabsorption) and urinary pH making it plausible that NHE3 transporter in the proximal tubule is indeed involved (reference). After prolonged administration, however, this natriuretic effect disappears as the kidney quickly adjust tubular sodium transport to match sodium excretion to sodium intake [54]. If enhanced sodium excretion would contribute to the cardiovascular and renal effects of GLP-1 receptor agonists, one would expect a reduction in plasma volume (or as a proxy: increased hematocrit) [55] or total body sodium content [56]. This seems however not to be the case and makes the role for altered sodium homeostasis unlikely to account for the renal and cardiovascular effects of GLP 1 receptor agonism.

### Hyperfiltration

Glomerular hyperfiltration is increasingly recognized as a risk factor that drives the progression of DKD. This can present either as elevated whole-kidney GFR in people with preserved nephron mass (e.g. in young individuals with early disease) as well as single-nephron hyperfiltration in older people with reduced nephron mass and/or later stages of disease [57]. Drugs that alter glomerular hyperfiltration such as RAS blockers and SGLT-2 inhibitors [58, 59] are among the most powerful drugs to prevent and reduce DKD burden. Given the effects of GLP-1 receptor agonists on proximal tubular sodium handling, in analogy with SGLT2 inhibitors, they could potentially alter renal hemodynamics by activating TGF. An early study by Gutzwiller et al. in hyperfiltering patients few of which had diabetes [60] as well as an open-label, uncontrolled study with liraglutide [61] suggested reductions in (estimated) GFR derived from creatinine clearance and ^51^Cr-EDTA, respectively. Several subsequent studies have more comprehensively detailed the effects of GLP-1 receptor agonism on glomerular hemodynamics using state-of-the-art inulin and para-aminohippuric acid (PAH) clearance techniques to measure GFR and effective renal plasma flow (ERPF), respectively. In contrast to the results of earlier studies, exenatide infusion increased GFR, ERPF and estimated glomerular hydraulic pressure in overweight but otherwise healthy men [51]. This increment in GFR and ERPF seemed to be caused by a reduction in estimated afferent renal arteriolar resistance, which was partly dependent on nitric oxide (NO). Subsequent studies with similar design showed no effect of acute or prolonged exenatide or liraglutide treatment on renal hemodynamics in people with T2D with or without DKD [52–54].

Thus, at present there is insufficient evidence to support attenuation of glomerular hyperfiltration in response to GLP-1 receptor agonists, as seems the case for RAS blockers or SGLT2 inhibitors.

### Inflammation

In recent years, the role of the immune system in the pathogenesis of DKD has received much attention. Kidney biopsies derived from people with DKD have shown that different cells of the immune system, most notably classically-activated macrophages and T-cells, accumulate in the kidney and interact with resident kidney cells [62]. In addition, both centrally and locally produced pro-inflammatory cytokines and chemokines, including tumor necrosis factor (TNF)-alpha, monocyte chemoattractant protein-1 (MCP-1/CCL2), various interleukins (IL-6 and IL-1β) as well as other cytokines under control of the master regulator nuclear factor kappa beta (NF-κβ) are increased in people with DKD. Increasing evidence shows that both pro-inflammatory and anti-inflammatory stimuli modulate GLP-1 secretion, while GLP-1 in turn regulates the immune system, both at the level of the kidneys and blood vessels [63]. In several preclinical studies, GLP-1 receptor agonists lowered the systemic levels of the pro-inflammatory cytokines, inhibited pro-inflammatory signaling pathways independent of glucose-lowering while concomitantly improving histological features of DKD [19]. This reduction in inflammatory tone could certainly contribute to improved renal outcomes and has also been speculated to underlie the cardiovascular benefits of GLP-1 receptor agonists. However, clinical studies are needed to elucidate these potential inflammatory pathways in response to GLP-1 receptor agonists.

### Renal hypoxia

Finally, renal hypoxia has been proposed to be a unifying pathway for most etiologies of CKD, including DKD [15]. People with DKD are prone to chronic hypoxia due to the combination of reduced oxygen supply through microvascular damage, excessive renal energy expenditure due to hyperglycemia-related glomerular hyperfiltration and associated increased sodium reabsorption, and a less oxygen efficient fuel profile. The less oxygen-efficient fuel profile observed in people with T2D is thought to be secondary to insulin resistance and mitochondrial dysfunction altering substrate metabolism as indicated previously. Based on their effects on endothelial function, GLP-1 receptors could improve renal vascularization and oxygen supply, whilst lowering oxygen consumption by attenuating in obesity-related hyperfiltration. On the other end of the equation, GLP-1 induced changes in substrate metabolism through improvements in insulin sensitivity and mitochondrial dysfunction could enhance ATP production, as was suggested in a study in rodents [48]. It is currently unclear whether GLP-1 receptor agonists modulate oxygen physiology in humans and whether they could ameliorate renal hypoxia. Mechanistic trials are needed to evaluate whether GLP-1 receptor agonists can correct the potential metabolic imbalance between increased renal energy expenditure and impaired substrate metabolism proposed to underlie DKD.

## Conclusion and future perspectives

In recent years, GLP-1 receptor agonists have received a central position in the management of hyperglycemia in people with T2D given their potent glucose-lowering actions. In addition, they improve blood pressure, body weight and dyslipidemia and are consistently shown to reduce CVD in a high-risk T2D population. The GLP-1 receptor agonists are safe to use in people with DKD, however, whether they are truly nephroprotective remains to be seen. Trials have shown that GLP-1 receptor agonists lower albuminuria, however it is uncertain whether this will translate into improvements in hard renal outcomes. Similarly, the small changes in eGFR trajectories (i.e. subtle reductions in eGFR decline versus placebo or titrated insulin) do not necessarily indicate renoprotection. As the GLP-1 receptor agonists induce an initial upsurge in eGFR with similar trajectories over time, this pattern may not indicate a reduction of glomerular pressure. The answers to these outstanding gaps in knowledge are currently addressed in the FLOW trial (Semaglutide on the Progression of Renal Impairment in Subjects With Type 2 Diabetes and Chronic Kidney Disease; NCT03819153). In this trial, the effects of once weekly subcutaneous semaglutide in patients with macro-albuminuria and impaired eGFR are examined over 5 years of follow-up. The primary endpoint is a composite of persistent eGFR decline of > 50%, reaching ESKD, death from kidney disease or death from CVD. Given the recent results of the CREDENCE trial, many patients will also be treated with an SGLT2-inhibitor, allowing the trial to study the combined use of these agents on renal outcomes. Of note, in people with advanced DKD, additional glucose-lowering beyond SGLT2 inhibition will be necessary, making GLP-1 receptor agonists valuable drugs in this population. Finally, the FLOW trial may shed further light on the potential mechanisms involved on how GLP-1 receptor agonism could alter renal physiology, combined with future mechanistic studies that aim to find proof for the effects of GLP-1 receptor agonists on the renal risk factors/pathophysiological mechanisms related to DKD as summarized in this review.
